# Metabolically active angiosperms survive passage through the digestive tract of a large-bodied waterbird

**DOI:** 10.1098/rsos.230090

**Published:** 2023-03-22

**Authors:** Simona Paolacci, Marcel A. K. Jansen, Vlastimil Stejskal, Thomas C. Kelly, Neil E. Coughlan

**Affiliations:** ^1^ School of Biological, Earth and Environmental Sciences and Environmental Research Institute, University College Cork, Distillery Fields, North Mall, Ireland, T23 TK30; ^2^ Faculty of Fisheries and Protection of Waters, South Bohemian Research Center of Aquaculture and Biodiversity of Hydrocenoses, Institute of Aquaculture, University of South Bohemia in Ceske Budejovice, Husova třida 458/102, 370 05, České Budějovice, Czech Republic

**Keywords:** Anatidae, avian vectors, duckweed, endozoochory, plant dispersal, vegetative propagule

## Abstract

Avian vectors, such as ducks, swans and geese, are important dispersers of plant propagules. Until recently, it was thought that small vegetative propagules were reliant on adherence to vectors and are unlikely to survive passage through the avian digestive tract. Here, we conclusively demonstrate that metabolically active angiosperms can survive passage through the digestive tract of a large-bodied waterbird. In addition, we show that extended periods of air exposure for up to 7 days does not inhibit the survival of plantlets embedded in faecal matter. Following air exposure, plantlets (*n* = 3000) were recovered from 75 faecal samples of mute swans, *Cygnus olor*, with the survival of 203 plantlets. The number of recovered and surviving plantlets did not significantly differ among durations of air exposure. For recovered plantlets, the long-term viability and clonal reproduction of two duckweed species, *Lemna minor* and *L. gibba*, were confirmed following greater than eight months of growth. These data further amplify the key role of waterbirds as vectors for aquatic plant dispersal and demonstrate the internal transport (i.e. endozoochory) of metabolically active plantlets. These data suggest dispersal of vegetative plant propagules by avian vectors is likely to be a common occurrence, underpinning connectivity, range expansion and invasions of some aquatic plants.

## 1. Introduction

Dispersal is an essential ecological process that underpins biological connectivity, as well as population and metacommunity dynamics [1–3]. Intriguingly, freshwater plants frequently occur in isolated aquatic habitats and show broader distributions than their terrestrial counterparts [4]. While directional water currents (i.e. hydrochory) can disperse propagules within freshwater systems, overland transport among hydrologically unconnected sites is facilitated by a variety of different animal and anthropogenic vectors [5–7]. In particular, animal-mediated dispersal (i.e. zoochory) is frequently considered to be the means by which aquatic plants are dispersed among unconnected waterbodies (e.g. [8]). Epizoochory (synonyms ectozoochory and exozoochory) consists of propagule dispersal via their adherence to the external surfaces of animals [9], while endozoochory is the transportation of propagules within animal vectors, by passage through the digestive tract or by regurgitation [10]. Accordingly, endozoochorous dispersal is typically associated with propagule structures that can survive the harsh mechanical and chemical forces of the avian digestive tract [11].

Duckweeds (i.e. Lemnaceae) are a family of floating freshwater monocotyledonous plants [12] that can be found inhabiting lakes, ponds and brackish water bodies across all continents, except Antarctica [13]. Presently, there are 36 known species of duckweed, divided into five genera (*Lemna*, *Spirodela*, *Landoltia*, *Wolffia* and *Wolfiella*) [14], several of which have spread beyond their native range and show invasive tendencies. Duckweed plantlets are characterized by a reduced body size and tend to consist of several fronds (i.e. small leaf-like structures) that range between less than 1 mm to 15 mm in size [15]. Depending on the species, fronds may carry one or more short roots (typically less than 30 mm), while others are rootless. Rapid growth by clonal propagation is characteristic of this family [12], while flowering and seed production are rare for nearly all species [13,16,17] Although zoochory is frequently considered a means by which duckweed species are dispersed among hydrologically unconnected waterbodies (e.g. [12]), epizoochory rather than endozoochory is thought to be the primary mechanism of dispersal [18]. As duckweeds are entirely composed of soft vegetative structures, mostly photosynthetic tissues with stomatal pores, it is generally surmised that duckweed plantlets are readily digested and therefore cannot survive gut passage.

Recently, Silva *et al*. [19] reported the extraction of 18 living *Wolffia columbiana* fronds from fresh faecal samples (*n* = 3) obtained from white-faced whistling ducks, *Dendrocygna viduata*, residing within a Brazilian wetland. This is an important finding as it is the first and only report of a whole duckweed surviving gut passage through the digestive tract of a bird. However, *W. columbiana* can assume two different physiological states: a rapidly growing state and a starch-rich survival state (i.e. a turion). Turions are considered to play a key role in survival during suboptimal conditions, as well as during the colonization of new habitats [20]. Turions are a specialized stress-survival stage, and their survival of gut passage would resemble the well-known passage through the guts of seeds of a wide variety of plant species. Turion and growing states of the rootless duckweed *W. columbiana* appear deceptively similar, and quantification of starch content and/or histological examination are the only ways to unambiguously distinguish between these two life stages [20]. While Silva *et al*. [19] state that only intact *Wolffia* plantlets, with resemblance to live plants (i.e. with a bright green colour and integral structure) were considered, this does not conclusively exclude the possibility that *Wolffia* plantlets were originally in a dormant, stress-resistant turion state when ingested by waterbirds. Thus, the question remains whether rapidly growing, metabolically active duckweed plants, and indeed metabolically active fragments of any macrophyte, can survive passage through the gut of a bird. Furthermore, *W. columbiana* fronds are particularly small (typically around 0.5–1.0 mm) and it remains unknown whether the findings by Silva *et al*. [19] can be extrapolated to larger aquatic plants.

Following gut passage, plantlets embedded within faecal matter may not immediately re-enter the aquatic environments, as waterbirds will frequently defecate in loafing, roosting and nesting sites immediately adjacent to waterbodies. Accordingly, it is possible that plantlets may remain embedded within faecal matter for several days prior to re-entering the waterbody via a chance physical disturbance (e.g. pushed or washed into a waterbody). Although it has been established that greater resistance to desiccation tends to increase survival and viability of vegetative plantlets and fragments [21], it remains unknown if encapsulation within faecal matter will aid or inhibit desiccation.

The present study reports the survival and long-term viability of two duckweed species belonging to the genus *Lemna* (i.e. *L. minor* and *L. gibba*) following the passage of fronds through the digestive tract of mute swans, *Cygnus olor*, coupled with extended periods of post-dispersal air exposure for up to 7 days. We hypothesized that air exposure would reduce the survival of duckweed plantlets embedded within swan faecal samples. Neither of these duckweed species produce true, stress-resistant turions [22]. These data show that actively growing vegetative propagules can survive passage through the gut of a large-bodied waterbird and thus amplify a new perspective for plant colonization pathways among aquatic environments.

## 2. Materials and methods

Mute swans, *Cygnus olor*, faecal samples were collected between October and November 2020 from a single site located in central Ireland (53°16′30.7′′ N, 7°12′30.9′′ W). Samples were collected from patches of grassland surrounding a multi-trophic aquaculture system used to produce rainbow trout, *Oncorhynchus mykiss*, and European perch, *Perca fluvialis*. The system included 16 canals that supported a mixed population of the duckweed species *Lemna gibba* (greater than 90%) and *Lemna minor* (less than 10%). This system is described in detail by Stejskal *et al*. [23]. Samples were collected following incidental observation of a breeding pair of *Cygnus olor* and their cygnets foraging and apparently ingesting duckweed. A breeding pair of *Cygnus olor* has inhabited the canals during the breeding season for over a 3-year period.

**Figure 1.**
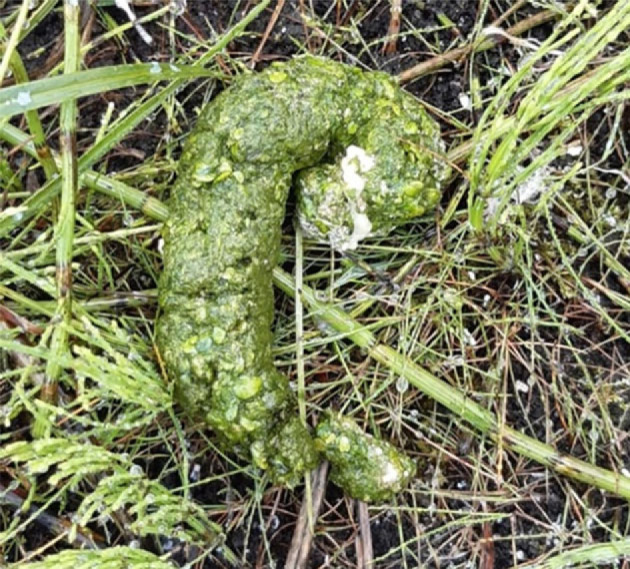
A representative example of the *Cygnus olor* faecal samples collected by this study.

In total, 75 faecal samples were collected across 13 different sampling days (figure 1). Each sample was individually placed into a sealable plastic bag. Between 3 and 10 fresh samples were collected on each sampling day, depending on sample availability, and immediately transported to a laboratory at University College Cork, Ireland.

### 2.1. Laboratory analysis

In the laboratory, the faecal samples were carefully extracted from the plastic bags, weighed and placed on shallow plastic trays (1.5 cm in depth) for air exposure. To assess the out-of-water survival of any encapsulated duckweed, the samples were exposed to a constant temperature (18 ± 2°C) and relative humidity regime (40–50% RH) for up to 7 days. These conditions were used to mimic the moderate to warm but damp conditions that are generally experienced during Irish summer months (June–August). As duckweed tends to be most abundant over the summer period, it is inferred that any ingestion and passive dispersal of duckweed will similarly be more likely to occur during periods of greater abundance.

Following air exposure, each faecal sample was individually placed in deeper (3.5 cm) plastic trays. Distilled water was then added to aid the gentle disintegration of each faecal sample. This process was carefully assisted with the aid of a stainless-steel micro-spatula, to tease apart the sample. When each sample had been turned into liquid suspension, duckweed fronds that maintained buoyancy on the surface were removed and counted. A single duckweed plantlet can be composed of one or more fronds (i.e. small leaf-like structures). Only floating fronds were considered, so as to exclude the possibility of survival through starch accumulating ‘heavy fronds’ known to occasionally occur in several duckweed species, which show reduced photosynthetic activity and sink to the bottom of waterbodies [22]. Further, to confirm the metabolic activity of floating fronds, the quantum yield of photochemical energy conversion in photosystem II was recorded (i.e. Y(II)). The quantum yield is an indicator of the efficiency of the photosynthetic light reactions under steady-state conditions, and it was measured for each frond using a FluorPen (Photon Systems Instruments, Drásov, Czech Republic). The recovered fronds were then moved into magenta vessels (GA-7, 7.7 cm length × 7.7 cm width × 9.7 cm height), containing 100 ml of half-strength Hutner's medium [24]). Plants were cultivated in a controlled growth room at 20°C, with a light intensity of 40 µmol m^−2^ s^−1^ and a light to dark photoperiod of 14 h : 10 h. After 7 days, the number of fronds still surviving was recorded. Etiolated fronds were considered dead, while fronds that appeared green and healthy were counted, with Y(II) being assessed once more to confirm metabolic activity.

Surviving fronds were maintained for approximately six months in magentas within the controlled growth room, with fresh Hutner's medium being changed on an ad hoc basis. In early May 2021, all duckweed biomass was placed in artificial, outdoor mesocosms (45 cm diameter, 30 cm depth). This was done to promote the expression of gibbosity (a convex ventral surface due to enlarged air chamber) in *L. gibba* to distinguish *L. gibba* from *L. minor* [25]. Under optimal laboratory conditions, the vegetative fronds of *L. minor* and *L. gibba* can appear morphologically identical.

### 2.2. Statistical analyses

Statistical analyses were conducted using R software ([26]; R 4.1.2). Logistic regression in the form of generalized linear models (GLM; car) was employed due to non-normal data and/or heteroscedastic residuals. The effect of the duration of air exposure was considered for the number of duckweed fronds recovered per gram of faecal sample, as well as the number of fronds that survived. Overall model significance was determined using likelihood ratio tests with *α* < 0.05 (lmtest).

## 3. Results

**Figure 2.**
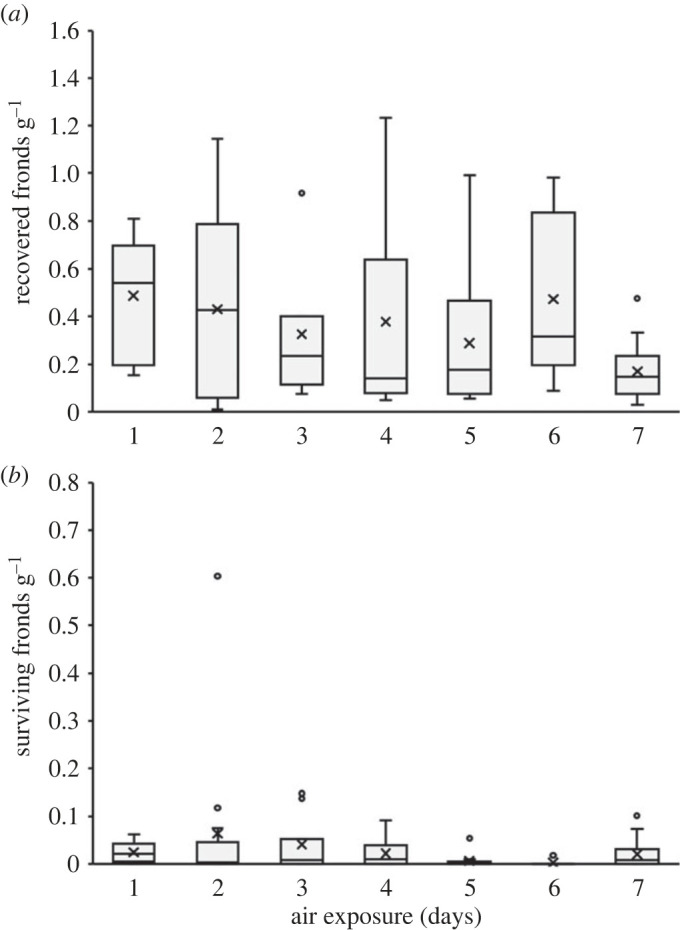
Median values for duckweed fronds recovered per gram of faecal sample (*a*), as well as frond survival following cultivation under optimal conditions for 7 days (*b*). Interquartile range (IQR), maximum and minimum IQR values, and outliers are shown, while cross (×) denotes mean values. Both recovered frond and viable frond numbers were statistically similar among exposure days (i.e. all *p* > 0.05).

Following air exposure, the number of fronds recovered ranged from 1 to 160 per faecal sample (40.0 ± 4.5, 27; mean ± s.e., median; electronic supplementary material, appendix S1). The number of fronds recovered per gram of sample did not vary significantly (GLM: *χ*^2^ = 12.062, d.f. = 6; *p* = 0.06; figure 2*a*). Similarly, following the 7-day assessment period for survival, the number of fronds surviving per gram of faecal sample did not significantly differ (GLM: *χ*^2^ = 7.4454, d.f. = 6; *p* = 0.28; figure 2*b*). Overall, approximately 93% of fronds originally recovered from samples had to some extent etiolated upon completion of the 7-day assessment period. However, most of the surviving fronds also displayed viability, as small, newly developed ‘daughter’ fronds were observed. Although Y(II) values were initially very low in fronds recovered from faecal samples (less than 0.04), indicating substantially impaired photosynthetic activity, values in surviving fronds bounced back to a value indicative of healthy fronds (greater than 0.6) (table 1). Duckweed plantlets were consistently observed to grow well over the approximately six-month indoor cultivation periods. Following the transfer of all duckweed cultures to outdoor mesocosms in early May, gibbosity of plantlets was observed by the end of June. Overall, *L. gibba* could be clearly identified within the mesocosms, with *L. minor* fronds without gibbosity also being present. However, the relative abundance was not quantified, as it would have been influenced by competition dynamics within the mesocosms.

**Table 1.** Duckweed fronds recovered from *Cygnus olor* faecal samples following 1–7 days of air exposure. Only fronds that could maintain buoyancy were considered, with survival being assessed after a 7-day cultivation period. Y(II) is the quantum yield of photochemical energy conversion in photosystem II. Initial Y(II) values for all fronds recovered from faecal samples were less than 0.04. Sample mass was recorded prior to air exposure. Values are mean ± s.e., while total sample *n* = 75.

air exposure (days)	sample (*n*)	sample mass (g)	recovered fronds	surviving fronds	final Y(II)
1	6	147.87 ± 33.63	79.00 ± 26.91	2.50 ± 1.06	0.70 ± 0.05
2	14	96.28 ± 14.31	40.57 ± 10.00	4.78 ± 2.85	0.69 ± 0.02
3	11	127.26 ± 28.60	32.45 ± 7.94	4.36 ± 1.80	0.72 ± 0.03
4	9	93.29 ± 19.46	40.96 ± 14.73	2.22 ± 1.02	0.77 ± 0.02
5	12	117.00 ± 15.30	36.58 ± 10.66	0.58 ± 0.50	0.75 ± 0.02
6	7	154.77 ± 28.48	58.71 ± 12.19	0.28 ± 0.20	0.74 ± 0.03
7	16	104.15 ± 20.66	23.93 ± 7.40	2.75 ± 1.19	0.78 ± 0.01

## 4. Discussion

The present study unambiguously demonstrates that whole angiosperms can be dispersed by gut passage through waterbirds in field environments. In particular, the resumption of growth as well as long-term viability for two duckweed species is shown (greater than eight months), following gut passage and subsequent air exposure of the faecal samples for up to 7 days. These data agree with reports in the literature that indicate duckweed can survive in wet mud for several months [27], which suggests that desiccation can be inhibited if relative humidity remains high in the microclimate immediately surrounding embedded duckweed [6,7]. A damp environment was probably provided within the faecal samples. However, swan faecal samples are relatively large compared with those produced by other Anatidae, such as duck species, and reduced faecal mass might increase desiccation. Nonetheless, surviving duckweed fronds recovered from faecal samples were able to re-establish efficient photosynthetic activity, a characteristic of healthy plants [28]. Given that only buoyant fronds were selected for assessment, it is concluded that metabolically active fronds can retain viability following gut passage through a large-bodied waterbird (body mass: 8.5–12 kg), as well as subsequent extended periods of incubation within faecal samples prior to re-entering the aquatic environment. Nevertheless, future research should consider how varied levels of frond metabolic activity prior to ingestion by waterbirds influences survival and resumption of growth for duckweed plantlets following avian gut passage.

These data show that angiosperms much larger than *W. columbiana*, with a frond size 0.5–1.0 mm in comparison with fronds of 8.0–10.0 mm for both *L. minor* and *L. gibba*, can survive gut passage. Following the recovery of 18 living *W. columbiana*, it has been suggested that the especially small size of *W. columbiana* fronds could be advantageous for gut passage survival [19], as seeds with a smaller size and round shape may quickly ‘escape’ the mechanical forces of the avian digestive tract [29,30]. The survival of a single bryophyte fragment (greater than 125 µm) following passage through the gut of a medium-sized waterbird (mallard duck, *Anas platyrhynchos*; body mass: 0.7–1.6 kg) has also previously been evidenced [31], as has viability for an apparent fragmentary propagule of invasive *Crassula helmsii* (barnacle goose, *Branta leucopsis*; body mass: approximately 1.7 kg) [32]. When taken together, these studies indicate that endozoochorous dispersal of vegetative propagules by waterbirds can occur more readily than previously thought. In turn, this triggers the question of potential endozoochorous dispersal for fragments of other aquatic macrophytes, including invasive species such as *Azolla filiculoides*, *Elodea* spp., *L. minuta* and *Myriophyllum* spp. among others. It has recently been demonstrated by Coughlan *et al*. [33] that invasive *C. helmsii*, *E. canadensis*, and *Lagarosiphon major* can grow from small, vegetative fragments (i.e. 1 cm in total length) even when cultivated under less-than-optimal conditions. Although endozoochory of seeds and even macroinvertebrates by waterbirds have been widely reported (e.g. [8,10,34]), there remains a need to consider endozoochory as a possible means of dispersal for vegetative propagules of clonally reproducing invasive macrophytes, which may even include fragments of the invasive grasses such as *Puccinellia phryganodes* [8,35].

Although the relatively high abundance of both duckweed species at the study site undoubtedly supported a high rate of ingestion, duckweeds along with many other aquatic macrophytes are frequently found in high abundance across natural and anthropogenic waterbodies. Further, the diets of common Anatidae across Europe and North America, including *C. olor*, have been observed to contain a variety of macrophytes including duckweed spp., *Elodea* spp. and *Myriophyllum* spp. (e.g. [36,37]). Further, duckweed plantlets appear to contribute to a complex pattern of dietary niche partitioning among dabbling ducks residing in agricultural landscapes [38], with the duckweed *Spirodela polyrhiza* being more regularly ingested by small-bodied ducks rather than large-bodied duck species. Consequently, small-bodied dabbling ducks might tend to ingest and disperse duckweeds more frequently than larger individuals.

Largely sedentary in many regions, with most regional dispersal distances tending to be less than 35 km (i.e. 54% of movements; [39]), *C. olor* inhabit freshwater, brackish and saltwater habitats [34]. While *C. olor* residing in Ireland have been recorded to relocate by up to 250 km among sites, with swans moving between coastal region of the British and Irish Isles across the Irish Sea [39]. Further, with a capacity for flight speeds of over 70 km h^−1^, *C. olor* and other *Cygnus* spp. do participate in long-distance migratory movements, notably among countries surrounding the Baltic Sea [34]. The calculated average range of 100–500 km for flight distances over which endozoochory is likely to occur increases with Anatidae body mass due to the longer retention time and greater flight speeds by larger waterbirds (e.g. from teal, *Anas crecca*, to *C. olor*; 0.3 to 10.8 kg) [34]. However, other models indicate that larger Anatidae may have shorter gut retention times compared with smaller waterbird species (e.g. [40]). Nevertheless, multiple short-distance dispersal events leading to stepping-stone dispersal among habitats could result in range expansion over time [18], with small-bodied Anatidae potentially being important vectors of duckweed spp. given longer gut retention times, along with the high degree of daily mobility among aquatic sites (e.g. [41]).

While relatively little is known about zoochorous dispersal by true swans (i.e. *Cygnus* spp.), given their overall ecology it has been hypothesized that they may be exceptionally important vectors of plant dispersal [29]. To date, endozoochorous dispersal by *Cygnus* spp. has only been considered for black swan, *Cygnus atratus*, which have been found to disperse a variety seeds and invertebrate propagules in Australia [42]. The present study lends support to this theory, especially as zoochory of vegetative fragments/plantlets would allow for plant dispersal outside of restricted seed production periods [19,34]. Further, our data indicate that vegetative propagules embedded in faecal matter would not need to immediately re-enter the aquatic environment, with potential out-of-water survival for several days until washed/pushed into a waterbody, such as by heavy rain. Overall, it is concluded that endozoochorous dispersal of vegetative propagules by Anatidae can underpin introduction, connectivity and persistence of clonal species among waterbodies.

## Data Availability

The data are provided in the electronic supplementary material [43].
